# Specialist-led community-based cardio-kidney-metabolic-pulmonary care in high-risk individuals: multi-centre pragmatic prospective randomized open-label blinded endpoint trial

**DOI:** 10.1093/ehjopen/oeag169

**Published:** 2026-10-07

**Authors:** Ramesh Nadarajah, Ali Wahab, Ben Hurdus, Carl Simela, Tobin Joseph, Catherine Reynolds, Sheena Bennet, Mohammad Haris, Louise Clark, Chris Hayward, Jing Kang, Jianhua Wu, Chris P Gale

**Affiliations:** Leeds Institute of Data Analytics, University of Leeds, 6 Clarendon Way, Leeds LS2 9DA, UK; Leeds Institute for Cardiovascular and Metabolic Medicine, University of Leeds, 6 Clarendon Way, Leeds LS2 9DA, UK; Department of Cardiology, Leeds Teaching Hospitals NHS Trust, Leeds LS1 3EX, UK; Leeds Institute of Data Analytics, University of Leeds, 6 Clarendon Way, Leeds LS2 9DA, UK; Leeds Institute for Cardiovascular and Metabolic Medicine, University of Leeds, 6 Clarendon Way, Leeds LS2 9DA, UK; Department of Cardiology, Leeds Teaching Hospitals NHS Trust, Leeds LS1 3EX, UK; Leeds Institute of Data Analytics, University of Leeds, 6 Clarendon Way, Leeds LS2 9DA, UK; Leeds Institute for Cardiovascular and Metabolic Medicine, University of Leeds, 6 Clarendon Way, Leeds LS2 9DA, UK; Department of Cardiology, Leeds Teaching Hospitals NHS Trust, Leeds LS1 3EX, UK; Leeds Institute for Cardiovascular and Metabolic Medicine, University of Leeds, 6 Clarendon Way, Leeds LS2 9DA, UK; Department of Cardiology, Leeds Teaching Hospitals NHS Trust, Leeds LS1 3EX, UK; Leeds Institute of Data Analytics, University of Leeds, 6 Clarendon Way, Leeds LS2 9DA, UK; Leeds Institute for Cardiovascular and Metabolic Medicine, University of Leeds, 6 Clarendon Way, Leeds LS2 9DA, UK; Leeds Institute for Cardiovascular and Metabolic Medicine, University of Leeds, 6 Clarendon Way, Leeds LS2 9DA, UK; Leeds Institute for Cardiovascular and Metabolic Medicine, University of Leeds, 6 Clarendon Way, Leeds LS2 9DA, UK; Leeds Institute of Data Analytics, University of Leeds, 6 Clarendon Way, Leeds LS2 9DA, UK; Leeds Institute for Cardiovascular and Metabolic Medicine, University of Leeds, 6 Clarendon Way, Leeds LS2 9DA, UK; Department of Cardiology, Leeds Teaching Hospitals NHS Trust, Leeds LS1 3EX, UK; Department of Cardiology, Leeds Teaching Hospitals NHS Trust, Leeds LS1 3EX, UK; Leeds Institute of Data Analytics, University of Leeds, 6 Clarendon Way, Leeds LS2 9DA, UK; Leeds Institute for Cardiovascular and Metabolic Medicine, University of Leeds, 6 Clarendon Way, Leeds LS2 9DA, UK; Oral Clinical Research Unit, Faculty of Dentistry Oral Craniofacial Sciences, King’s College London, London, UK; Wolfson Institute of Population Health, Queen Mary, University of London, UK; Leeds Institute of Data Analytics, University of Leeds, 6 Clarendon Way, Leeds LS2 9DA, UK; Leeds Institute for Cardiovascular and Metabolic Medicine, University of Leeds, 6 Clarendon Way, Leeds LS2 9DA, UK; Department of Cardiology, Leeds Teaching Hospitals NHS Trust, Leeds LS1 3EX, UK

**Keywords:** Cardiovascular disease, Primary care, Prevention, Risk factor, Treatment, Randomized controlled trial

## Abstract

**Aims:**

Pharmacological therapies that treat cardiovascular, kidney, metabolic, and pulmonary (CKMP) risk factors reduce the risk of major adverse cardiovascular events, but community-dwelling individuals may not receive all treatments to which they are eligible from non-specialists. This trial investigated if specialist-led care in the community increases the utilization of CKMP pharmacological interventions compared with usual care.

**Methods and results:**

This pragmatic prospective randomized open-label blinded endpoint trial included community-dwelling high-risk adults aged ≥30 years from 15 primary care sites in England. Participants were randomized 1:1 to an intervention of two clinic appointments (at baseline and ∼3 months) with a cardiology specialist at their primary care site to review and optimize CKMP risk factors in accordance with UK guidelines, or usual care. The primary outcome was the proportion of participants with initiation and/or increase in the dose of guideline-directed CKMP pharmacological therapies at 6 months after date of randomization. Between March 2025 and July 2025, 138 participants were randomized (68 intervention, 70 control); the median age was 76.6 years, and 52.9% were women. At 6 months after randomization, in the intervention arm, 42 of 68 (61.8%) patients received a CKMP pharmacological intervention compared with 16 of 70 patients (22.9%) in the control arm (odds ratio 5.4, 95% CI 2.5–12.3, *P* < 0.0001, absolute risk difference 38.9%, 95% CI 23.7–54.1%).

**Conclusion:**

Specialist-led care in the community for high-risk individuals increased the use of pharmacological interventions for CKMP risk factors compared to usual care.

## Graphical Abstract

**Graphical Abstract:**
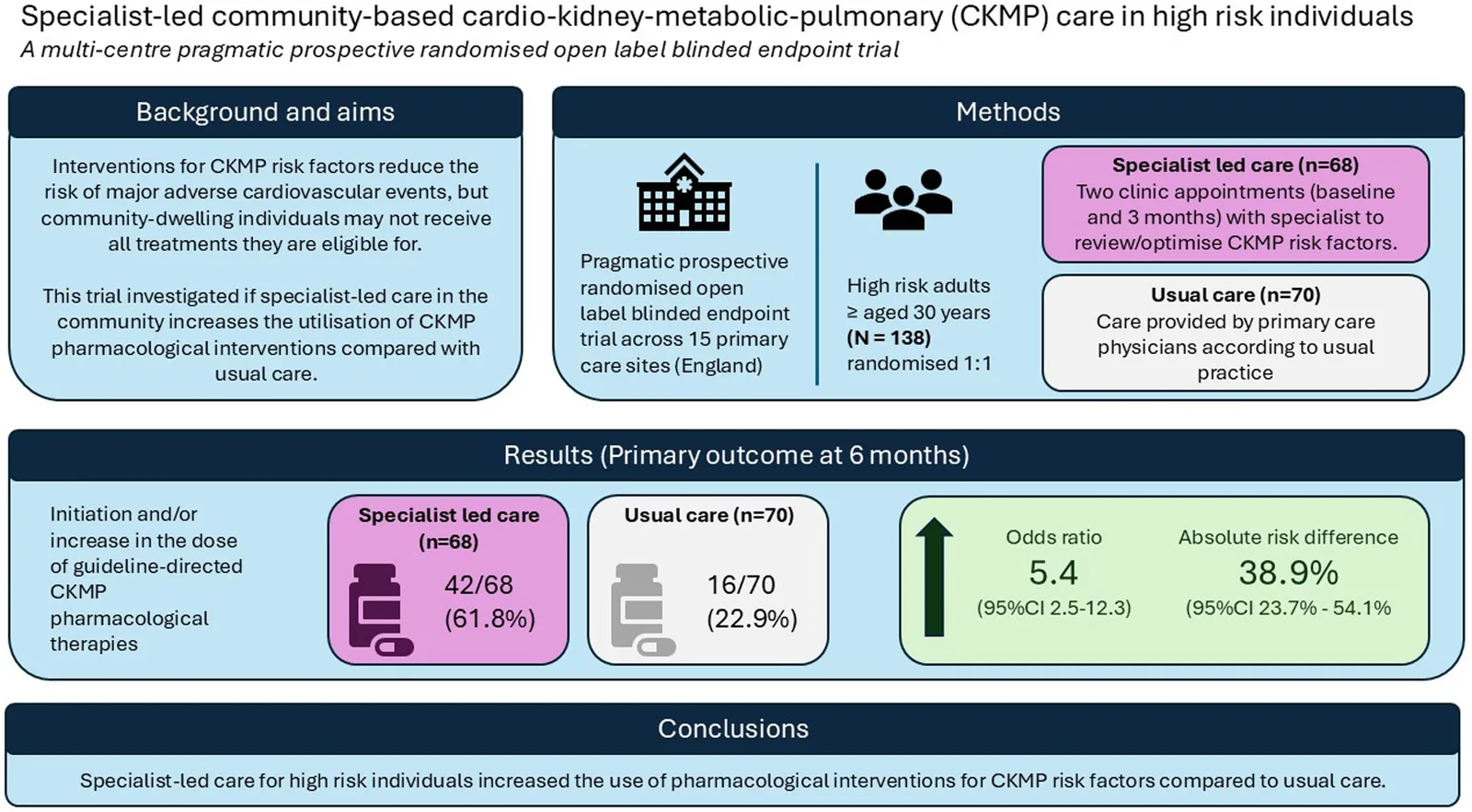

## Introduction

Treatment of hypertension, dyslipidaemia, diabetes mellitus, chronic kidney disease (CKD), and obesity has been shown in randomized controlled trials to reduce the risk of cardiovascular disease and cardiovascular death.^1–6^ Guidelines recommend pharmacological interventions across cardiovascular, kidney, metabolic, and pulmonary (CKMP) risk factors to reduce the risk of major adverse cardiovascular events.^6–11^

Not prescribing guideline-directed medical therapies for CKMP risk factors, or not increasing their dose as recommended by guidelines, may increase the risk for major adverse cardiovascular events. Yet, it is increasingly complex and challenging, given the clustering of CKMP risk factors in contemporary patients, to ensure that an individual in the community is in receipt of the most appropriate medications at the correct doses. Our prior work has suggested that high-risk community-dwelling individuals could receive more pharmacological intervention to optimize CKMP risk factors in accordance with guidelines.^12^

It is hypothesized that modern healthcare pathways should be more integrated and specialists involved in chronic community-based management of CKMP risk factors to reduce the risk of major adverse cardiovascular events.^13^ In the OPTIMISE-1 RCT, we sought to test the effect of community-based specialist-led care on the utilization of therapeutic interventions for CKMP risk factors compared to usual care among high-risk, community-dwelling, individuals.

## Methods

### Study design

This multi-centred pragmatic prospective randomized open-label blinded-endpoint (PROBE) RCT was conducted at 15 primary care sites. The study protocol was approved by the North East & North Tyneside 2 Research Ethics Committee (reference 24/NE/0188). Details of the trial design have been described previously.^14^ The trial conformed to the Declaration of Helsinki, and the reporting of results was based on the CONSORT (consolidated standards of reporting trials) statement.^15^ The trial was registered with ClinicalTrials.gov (NCT06444711).

### Participants

Eligible individuals were aged ≥30 years and at high risk for CKMP diseases and cardiovascular death based on the FIND-AF risk stratification tool in their electronic health records. Participants were recruited from a longitudinal consented FIND-AF cohort who had provided consent for invitation to future studies.^16,17^ The FIND-AF cohort (including 1923 community-dwelling participants) were originally recruited into the FIND-AF study to undergo atrial fibrillation (AF) screening, and the cohort for RCT recruitment includes both patients with and without diagnosed AF. FIND-AF is a random forest multivariable prediction model trained to predict AF within a 6-month prediction horizon. The variables included in FIND-AF were restricted to age, sex, and comorbidities, which means it can be scaled across primary care electronic health records. A FIND-AF score of ≥0.0053 is the threshold to be defined as high risk. Previous studies have shown that high FIND-AF risk is associated with increased risk of incident heart failure, stroke, myocardial infarction, and peripheral vascular disease, and the high-risk group constitutes 74% of incident cardiovascular or cerebrovascular deaths over 10-year follow-up (more details in the Supplementary material).^18^

Exclusion criteria included inability to give written informed consent for participation in the study, inability to adhere to the study requirements, and presence on the palliative care register.

### Randomization and masking

Participants were randomly assigned 1:1 to receive specialist-led care or usual care. Randomization was completed centrally on the OPTIMISE1 RCT randomization app, which is held on university computers at the Leeds Institute of Data Analytics. Randomization used stratification by three factors: age (>75 years and ≤ 75 years), sex (man and woman), and risk score (FIND-AF score > 0.01005 and ≤0.01005). Recruiting healthcare professionals were unaware which arm the participant would be randomized to. The treatment group assignment was known to the investigators and patients (open label), but this was masked to the endpoint adjudication committee members (T.J., M.H.), who reviewed the results of participants without access to information about allocation.

### Procedures

Participants in the specialist-led care group received a community-based appointment with a cardiology specialist at their primary care network at as close to baseline and at 3 months after randomization as possible. At the first appointment, they underwent observations, cardiac auscultation, spirometry, and review of laboratory tests for cardio-kidney-metabolic risk factors, or had these laboratory measures taken if not available and indicated according to National Institute for Health and Care Excellence (NICE) guidelines.^8–11,17,19,20^ Based on this assessment, the specialist recommended alterations to CKMP care in accordance with NICE guidelines to the participant and their GP, and/or the diagnosis of a CKMP risk factor. The specialist did not prescribe medications or add diagnostic codes directly; this was only recommended to the participant’s GP by way of a new task message inserted on the primary care electronic health record system. The second appointment was preferentially delivered in-person but was also offered by telephone to suit each participant’s wishes.

Control arm participants had their records reviewed at baseline to extract data on CKMP risk factors and prescribed medication but were not seen and no recommendations were made. Their clinical management followed ‘usual care’, as determined by their primary care physician.

At 6 months after randomization, primary care electronic health records of all participants were reviewed in control and intervention arms to extract primary and secondary outcome data by research team members masked to treatment group assignment (B.H., C.S.).

### Outcomes

The primary outcome was the proportion of patients with initiation and/or increase in the dose of any of the therapeutic interventions to optimize CKMP disease and/or risk factor management including

- Renin-angiotensin system inhibitors with multiple indications: angiotensin converting enzyme inhibitor/angiotensin 2 receptor blocker
- Hypertension management: calcium channel blocker/thiazide-like diuretic/spironolactone/alpha blocker/beta-blocker
- Glycaemic agents with cardiovascular benefit: sodium glucose co-transporter 2 (SGLT2) inhibitor and glucagon-like peptide-1 receptor agonist (GLP-1 RA)
- Glycaemic agents without cardiovascular benefit: metformin/dipeptidyl peptidase 4 (DPP4) inhibitor/pioglitazone/sulphonylurea/insulin
- Lipid modification agents: statin/ezetimibe/icosapent ethyl/bempedoic acid/proprotein convertase subtilisin/kexin type 9 (PCSK9) inhibitor
- Obesity-specific treatment: orlistat/GLP-1 RA (liraglutide, semaglutide) or GLP-1 RA/gastric inhibitory peptide agonist (tirzepatide)
- Smoking cessation: nicotine replacement therapy/bupropion
- Respiratory inhaler therapy: short acting beta agonist/short acting muscarinic antagonist/long acting beta agonist/long acting muscarinic antagonist/inhaled corticosteroid—or combination inhalers

If multiple interventions occurred in a single participant, these were counted as a single event for the primary outcome.

The secondary outcomes were components of primary outcome, time to primary outcome, diagnosis of new CKMP risk factor (hypertension, diabetes, CKD, obesity, current smoking, chronic obstructive pulmonary disease), and time to diagnosis of a new CKMP risk factor between randomization and 6 months follow-up. The component of the primary outcome that GLP-1 RA prescription was assigned to was based on indication recorded in the primary care electronic health record.

### Statistical analysis

Analyses were conducted according to randomized treatment allocation. Baseline characteristics were summarized by trial arm using counts and percentages for categorical variables and means with standard deviations or medians with interquartile ranges (IQR) for continuous variables, as appropriate. No formal statistical testing of baseline characteristics was undertaken.

The primary outcome was treated as a composite binary outcome and analysed as intention-to-treat irrespective of compliance. For each trial arm, we reported the number and percentage of participants meeting the outcome. The primary comparison between intervention and control arms was estimated as an odds ratio with 95% confidence interval using logistic regression with randomized group as the only explanatory variable. The same approach was used for the individual components of the primary outcome and for new diagnosis of CKMP risk factors. Results were presented as event counts and percentages by arm, odds ratios, 95% confidence intervals, and two-sided *P*-values where appropriate.

In additional analyses of pharmacological interventions, we examined the number of medication initiations and/or dose increases per participant for the primary outcome and each individual primary outcome component. Multiple medication changes within the same participant were retained, including changes occurring at different time points and across different medication groups. For visualization, we produced patient-level heatmaps showing the timing of medication changes of all observed events from randomization to 180 days, stratified by randomized arm and outcome component.

The primary analysis used a two-sided significance threshold of *P* < 0.05. Secondary outcomes and component analyses were not adjusted for multiple comparisons and were interpreted as exploratory. Analyses were performed using R version 4.6.0.

### Sample size

Based on our previous work, we estimated that the management of CKMP risk factors would be between 33 and 65% across different components (hypertension, diabetes etc.).^21^ Thus, we estimated that 40% of the intervention group would meet the primary outcome, that 10% would meet the primary outcome in the control group, and there would be 5% dropout given the short duration of the trial. A sample size of 138 participants randomized 1:1 to intervention and control, provided in excess of 90% power to detect the absolute difference of 30% with α = 0.05.

## Results

Between the 27 March and 31 July 2025, 205 patients were invited, and 138 enrolled and randomly assigned to specialist-led care (*n* = 68) or usual care (*n* = 70) (*Figure 1*). Participants came from 15 primary care sites (see Supplementary material online, *Table S1*). The date of 6 months follow-up for the final patient in was 31 January 2026. Of the 138 randomized participants, mean age was 76.6 years (standard deviation 5.7) and 73 (52.9%) were women (*Table 1*). Group baseline characteristics were well balanced though patients in the usual care arm had a higher prevalence of recorded CKD (*Table 1*). Baseline CKMP medications were well balanced (see Supplementary material online, *Table S2*).

**Figure 1:**
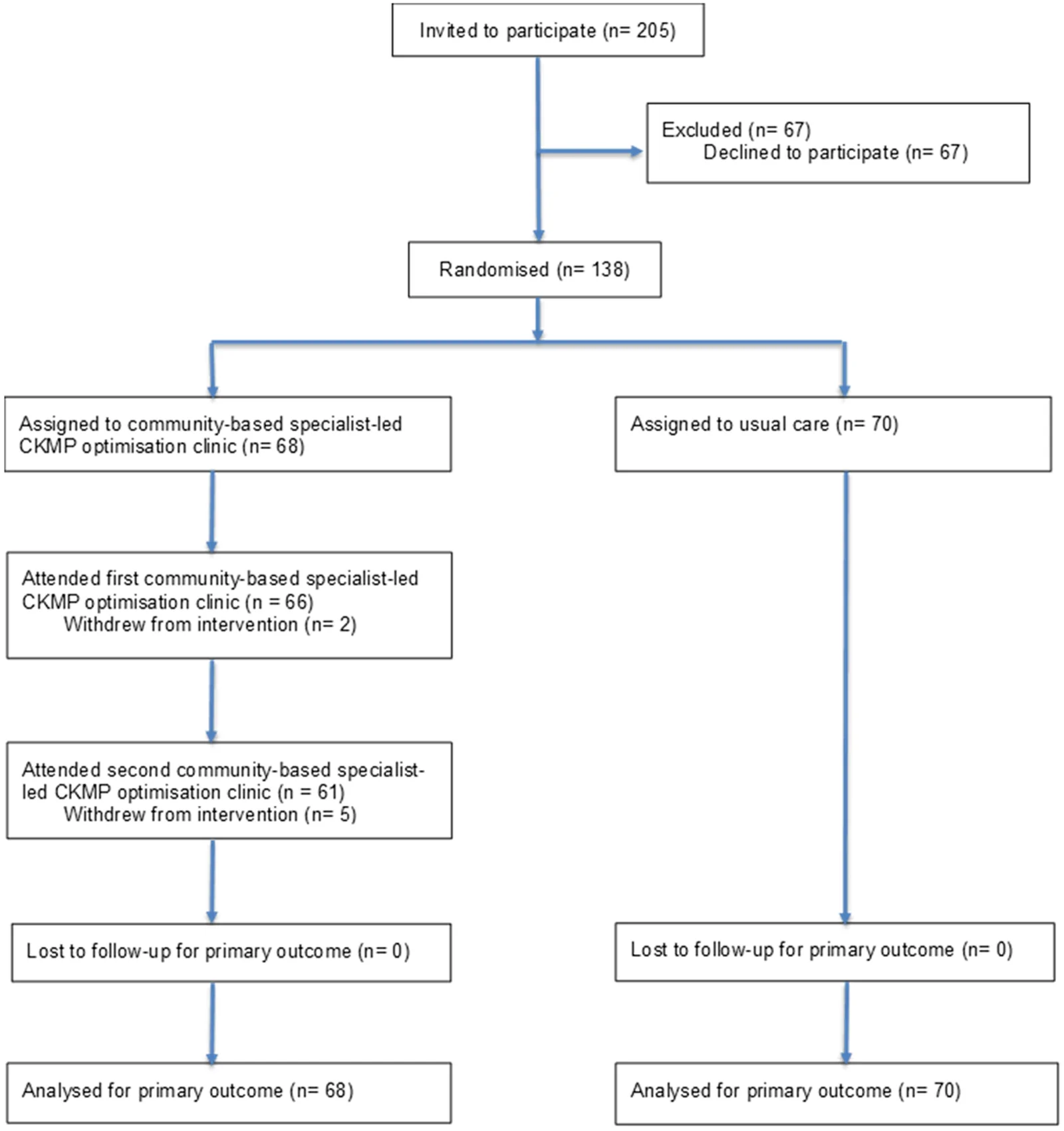
Flow of participants in the OPTIMISE-1 trial. CKMP, cardio-kidney-metabolic-pulmonary.

**Table 1:** Baseline characteristics of participants

Characteristics	Intervention (*n* = 68)	Control (*n* = 70)
Age (years), median (IQR)	76.6 (6.4)	76.5 (5.0)
Male sex	33 (48.5)	32 (45.7)
Ethnicity (White)	59 (86.8)	63 (90.0)
Atrial fibrillation	4 (5.9)	7 (10.0)
Chronic kidney disease	13 (19.1)	24 (34.3)
Diabetes mellitus	16 (23.5)	16 (22.9)
Dyslipidaemia	19 (27.9)	18 (25.7)
Heart failure	9 (13.2)	7 (10.0)
Hypertension	52 (76.5)	52 (74.3)
Ischaemic stroke or transient ischaemic attack	6 (9.1)	8 (11.4)
Ischaemic heart disease	16 (24.2)	14 (20.0)
Peripheral arterial disease	1 (1.5)	3 (4.3)
Smoking (current)	6 (8.8)	6 (8.6)

Data are numbers (%) unless otherwise stated.

In the intervention group, 66 (97.1%) attended the first CKMP optimization clinic visit, and 61 (89.7%) attended the second CKMP optimization clinic visit (*Figure 1*). All participants who attended the first CKMP optimization clinic attended in-person, and 50 (82.0%) of those who attended the second CKMP optimization clinic attended in-person. For the intervention arm, the median time from randomization to the first CKMP optimization clinic was 1.0 month (interquartile range 0.7–1.2 months), and the median time randomization to the second CKMP optimization clinic was 4.0 months (interquartile range 3.5–4.4 months). There was no loss to follow-up, and no patients died during follow-up; accordingly, there were no missing data for the primary or secondary outcomes. There was no cross-over from the control group to the community-based specialist-led care intervention.

### Primary outcome

At 6 months after randomization, in the intervention arm, 42 of 68 (61.8%) participants had a CKMP pharmacological intervention compared with 16 of 70 patients (22.9%) in the control arm (odds ratio 5.4, 95% CI 2.5–12.3, *P* < 0.0001, absolute risk difference 38.9%, 95% CI 23.7–54.1%) (*Figure 2*). The number of high-risk participants needed to attend a specialist-led community-based clinic for a CKMP pharmacological intervention was 2.6.

**Figure 2:**
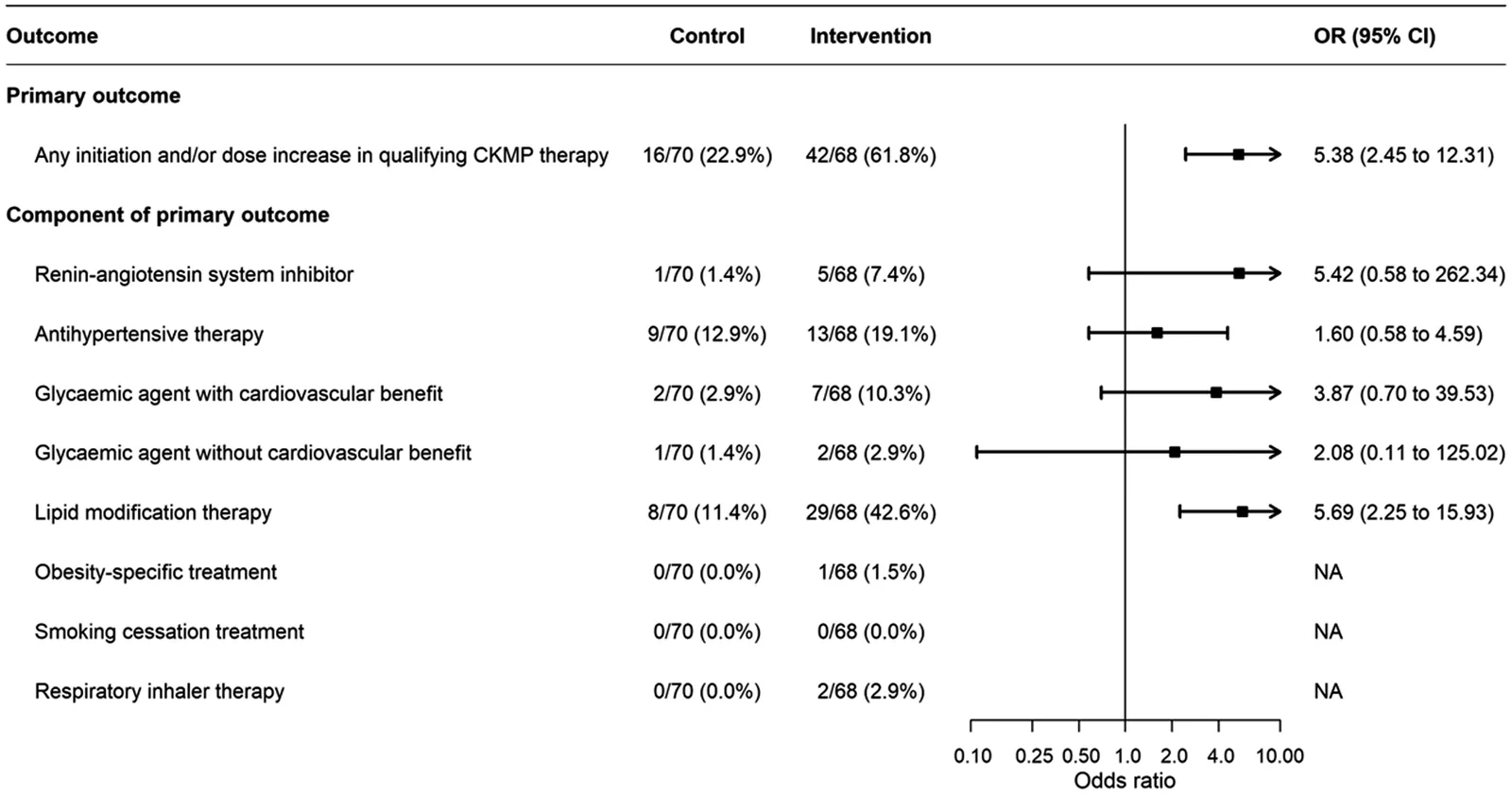
Forest plot showing effect of specialist-led care in the community on the primary outcome of cardio-kidney-metabolic-pulmonary pharmacological interventions and its individual components. CKMP, cardio-kidney-metabolic-pulmonary.

In total, in the intervention arm, there were 60 CKMP pharmacological interventions across 68 patients compared with 23 in 70 patients in the control arm, with 42 new prescriptions of CKMP medicines in the intervention arm compared with 17 new prescriptions in the control arm and 18 increases in CKMP medicine doses in the intervention arm compared with 6 increases in CKMP medicine doses in the control arm (see Supplementary material online, *Table S3*).

### Secondary outcomes

With regard to components of the primary outcome, at 6 months after randomization, 29 of 68 patients in the intervention arm had a lipid modification intervention compared with 8 of 70 patients in the control arm (odds ratio 5.7, 95% CI 2.3–15.9, absolute risk difference 31.2%, 95% CI 17.3–45.1%). The proportion of patients with a pharmacological intervention for each of the other CKMP components, except for smoking cessation therapy, was numerically higher for the intervention arm compared with the control arm, though these comparisons did not reach statistical significance (*Figure 2*).

In the intervention arm, 12 of 68 (17.6%) of patients had received a new diagnosis of a CKMP risk factor compared with 3 of 70 patients in the control arm (4.3%) (odds ratio 4.7, 95% CI 1.2–27.4, *P* = 0.014, absolute risk difference 13.4%, 95% CI 3.1–23.6%) (see Supplementary material online, *Figure S2*).

### Specialist-led recommendations uptake

In the intervention arm, of 81 pharmacological initiations or increases recommended during the specialist-led clinic, 47 (58.6%) were implemented by 6 months. Completion of recommended pharmacological interventions were 28 of 41 (68.3%) for lipid modification, 8 of 9 (88.9%) for antihypertensives, 6 of 6 (100%) for renin-angiotensin-aldosterone inhibitors, 4 of 8 (50.0%) for glycaemic agents with cardiovascular benefit, 0 of 4 (0%) for glycaemic agents without proven cardiovascular benefit, 1 of 12 (8.3%) for obesity medications, and 0 of 1 (0%) for smoking cessation therapy. Reasons for not following the recommendations are summarized in Supplementary material online, *Table S4*. For the prescription of obesity medications, 9 of the 11 (81.8%) recommendations were not followed due to discordance between NICE guidelines and local prescribing guidelines at the time of study delivery. Of 28 new CKMP diagnoses recommended to be added to the primary care electronic health records during the specialist-led clinic, 5 (17.9%) were recorded by 6 months. Of CKMP diagnoses recommended, 1 of 3 (33.3%) for CKD, 4 of 19 (21.1%) obesity, 0 of 1 (0%) for current smoking, and 0 of 5 (0%) for dyslipidaemia were coded in the primary care electronic health records at 6 months.

## Discussion

In this multi-centre pragmatic prospective randomized open-label blinded endpoint trial, community-based specialist-led consultations seeking to address CKMP risk factors significantly increased the proportion of patients who received an initiation or increase of a medication for a CKMP risk factor compared to usual care. Community-based specialist-led consultations also significantly increased the proportion of patients who received a diagnosis of a CKMP risk factor compared with usual care. The highest proportion of CKMP medicine increase or initiation was for lipid modification. Community-based specialist-led consultations frequently recommended the coding of obesity diagnoses and the treatment with GLP-1 receptor agonists in accordance with NICE guidelines, but this was infrequently actioned because of discordance with local prescribing guidelines.

Previous RCTs have demonstrated that structured interventions can improve control of cardiometabolic risk factors in community-dwelling individuals.^22–24^ In Canada, the SCRIP-HTN trial demonstrated that a community pharmacist–nurse intervention improved blood pressure control among patients with diabetes, while the larger RxEACH trial showed that pharmacist-led cardiovascular risk assessment, prescribing, and longitudinal follow-up reduced estimated cardiovascular risk and improved blood pressure, LDL cholesterol, and glycaemic control.^23,24^ In the USA, the TEAMcare trial demonstrated that nurse-led collaborative management, supported by specialist supervision and primary care physicians, improved glycaemic, lipid and blood pressure control in patients with diabetes and/or coronary heart disease.^22^ Collectively, these studies support the principle that additional specialist or multidisciplinary input can reduce therapeutic inertia in the community across different healthcare systems.

However, these RCTs were generally focussed on risk factor control through pharmacological and non-pharmacological approaches. Over recent years, a range of pharmacological classes, including SGLT2 inhibitors and incretin-based therapies, have become available in clinical practice which affect multiple components of CKMP pathways and are indicated for conditions across this axis (for example in heart failure, CKD, and diabetes), with RCT evidence for reducing the risk of major adverse cardiovascular events.^25,26^ Furthermore, it is hypothesized that these medicines are protective for major adverse cardiovascular events through mechanisms beyond improving control of glycated haemoglobin, delaying deterioration in renal function, or weight reduction.^4,27^ Accordingly, this RCT was designed with the hypothesis that preventative cardiovascular care should move beyond a narrow focus on optimizing individual risk factors and seek to increase the use of prognostically beneficial medicines across CKMP conditions. Furthermore, this RCT evaluated a pragmatic model in which specialist recommendations were delivered within primary care while prescribing remained with the primary care physician. The consistency of benefit across these different models supports the potential transferability of specialist-supported CKMP care beyond the UK, although the optimal mix of healthcare professionals, where prescription responsibilities sit, and how specialist-led advice should be provided are likely to depend on local healthcare organization and scope-of-practice arrangements.

While the intervention incorporated pharmacological interventions across the wider CKMP axis, lipid modification was the most common initiation or increase. Despite robust RCT evidence that lipid modification reduces the risk of major adverse cardiovascular events,^28^ incentivization to optimize lipid control in primary care in England has been relatively fragmented. Notably, in contrast to diabetes, heart failure, or pulmonary disease, there is a lack of primary care-based specialist nurse care for lipid modification across primary and secondary prevention. Obesity was recommended to be coded for over a quarter of intervention arm participants, and GLP-1 receptor agonists were recommended in accordance with NICE guidelines for almost a fifth of intervention arm participants, but these recommendations were infrequently actioned. This study was designed to be pragmatic^29^ and test the intervention of specialist expertise and review in the community, with the responsibility for starting and continuing medicines remaining with the primary care physician. This led to discordance between NICE guidelines for obesity and local availability of tiered specialist referral pathways and local prescribing commissioning. While NICE guidelines recommended prescription of semaglutide, tirzepatide, and liraglutide based on the presence of elevated body mass index, access to these medications was ringfenced at the time of this trial to weight management services, which were not accessible to practices participating in the study. The Obesity Pathway Innovation Programme seeks to evaluate new weight management care models including pharmacy-based and primary care partnerships,^30^ and several implementation models are being piloted to support prescribing of GLP-1 receptor agonists in primary care.^31^ Once these prescribing models have reached maturity, it is possible that the specialist-led community-based intervention for the CKMP axis tested in this RCT could be even more effective.

### Study limitations

Given the pragmatic trial design (and focussing on the primary outcome of medicine increase or initiation),^29^ control arm participants did not attend baseline or follow-up assessment of observations or laboratory tests thus precluding a randomized comparison of risk factor control. Also, the study did not seek to assess the effect of this intervention on clinical outcomes; the included pharmacological interventions for lipid modification, diabetes, CKD, and obesity have been demonstrated in RCTs to reduce major adverse cardiovascular events. Notably, the study was powered for the primary outcome and does not have statistical power for secondary outcomes, nor did it include a health economics analysis. Due to the small sample size we did not undertake sub-group analyses or undertake categorization according to receipt of ‘optimal care’ as specified in the statistical analyses section of the published protocol.^14^ Though this intervention incorporated a cardiology specialist, future implementation could involve a nurse- or pharmacist-led service with supervision from a cardiology specialist. Furthermore, direct prescription and diagnostic coding for CKMP medicines and risk factors (rather than recommendation to primary care) may improve implementation of medicines, as fewer than two-thirds of specialist-recommended pharmacological interventions were implemented by primary care healthcare professionals in this trial. Study participants were part of a longitudinal cohort-RCT platform originally formed as part of a screening study for AF,^16,17^ which may have led to a lower prevalence of AF among participants than the age- and sex-matched general population. Nonetheless, participants reflected an older and comorbid population, and this at-risk population has been shown to have high rates of incident CKMP diseases, cardiovascular death, and all-cause death in an earlier study.^18^ Of consented participants, 83 of 138 (60.1%) originated from two large primary care sites, with 55 of 138 (39.9%) of participants originating from thirteen other primary care sites. Though the intention was to randomize 1:1 to the intervention and control groups, it ended up that two extra participants were in the control group. However given the large effect observed for specialist-led consultations to increase the proportion of participants who received who received an initiation or increase of a medication for a CKMP risk factor compared to usual care, we do not believe this had an impact on the study findings.

## Conclusions

In conclusion, specialist-led community-based clinics focussed on the optimization of CKMP guideline-directed medical therapy increased the prescription of CKMP pharmacological therapies at 6 months compared with usual care. Increased involvement of specialists in the management of CKMP risk factors in the community could be implemented to optimize care with the aim of reducing downstream occurrence of major adverse cardiovascular events.

## Supplementary Material

oeag169_Supplementary_Data

## Data Availability

Anonymized data might be shared upon reasonable request.
