# Supplementary material for: Specialist-led community-based cardio-kidney-metabolic-pulmonary care in high-risk individuals: multi-centre pragmatic prospective randomized open-label blinded endpoint trial
Source: Eur Heart J Open. 2026 Oct 7;6(5):oeag169. doi: 10.1093/ehjopen/oeag169 (PMC13712192; doi:10.1093/ehjopen/oeag169)
Supplement: Supplementary file 1 [file oeag169_supplementary_data.docx]

**Supplementary Material**

Specialist-led community-based cardio-kidney-metabolic-pulmonary care in high risk individuals: multi-centre pragmatic prospective randomised open label blinded endpoint trial

[Supplementary Methods	2](#_Toc238510328)

[Identification of high risk community-dwelling individuals for cardio-kidney-metabolic-pulmonary disease.	2](#_Toc238510329)

[Supplementary Results	4](#_Toc238510330)

[Supplementary Table 1: Participating general practice sites and recruited participants	4](#_Toc238510331)

[Supplementary Table 2. Cardio-kidney-metabolic-pulmonary medications at baseline in the control and intervention group	5](#_Toc238510332)

[Supplementary Table 3. CKMP medication interventions by six months	6](#_Toc238510333)

[Supplementary Table 4. Reasons for non-adherence to CKMP medication recommendations	7](#_Toc238510334)

[Supplementary Figure 1. Heat map of cardio-kidney-metabolic-pulmonary pharmacological interventions	8](#_Toc238510335)

[Supplementary Figure 2. Forest plot of new cardio-kidney-metabolic-pulmonary risk factor diagnoses	9](#_Toc238510336)

[References	11](#_Toc238510337)

# Supplementary Methods

## Identification of high risk community-dwelling individuals for cardio-kidney-metabolic-pulmonary disease.

High risk individuals were recruited from the FIND-AF cohort-RCT platform who had consented to be approached about further studies. The FIND-AF cohort (including 1923 community-dwelling participants) were originally recruited into the FIND-AF study to undergo atrial fibrillation (AF) screening and the cohort for RCT recruitment includes both patients with and without diagnosed AF. FIND-AF is a random forest multivariable prediction model trained to predict AF within a 6-month prediction horizon. The variables included in FIND-AF were restricted to age, sex, and comorbidities, which means it can be scaled across primary care electronic health records. A FIND-AF score of ≥0.0053 is the threshold to be defined as high risk.

In an observational study using routinely collected UK primary care EHRs,^1^ we found high FIND-AF risk, compared with low FIND-AF risk, was associated with incident chronic obstructive pulmonary disease (cumulative incidence per 1000 persons at 10 years 395.8; hazard ratio 2.02, 95% CI 2.00-2.05; median time to event 2.68 years), chronic kidney disease (245.2; 6.85, 6.70-7.00; 5.44), heart failure (124.7; 12.54, 12.08-13.01; 4.06), diabetes mellitus (123.3; 2.05, 2.00-2.10; 3.45), stroke/transient ischaemic attack (118.9; 8.07, 7.80-8.34; 4.27), myocardial infarction (69.6; 5.02, 4.82-5.22; 4.32), peripheral vascular disease (44.6; 6.62, 6.28-6.98; 4.28), valvular heart disease (37.8; 6.49, 6.14-6.85; 4.54), aortic stenosis (18.7; 9.98, 9.16-10.87; 4.41), and death from any cause (273.9; 10.45, 10.23-10.68; 4.75). The high risk group constituted 74% of deaths from cardiovascular or cerebrovascular causes (8 582/11 676).

Accordingly high FIND-AF risk individuals were deemed at high risk of incident cardio-kidney-metabolic-pulmonary (CKMP) disease as well as cardiovascular death, and an appropriate population to test the intervention of specialist-led care compared to usual care in the community for utilisation of CKMP pharmacological interventions.

# Supplementary Results

## Supplementary Table 1: Participating general practice sites and recruited participants

| **Practice** | **Total** | **Control** | **Intervention** |
| --- | --- | --- | --- |
| Albion Mount medical practice | 41 | 20 | 21 |
| Calverley medical centre | 10 | 3 | 7 |
| Cowgill medical practice | 1 | 1 | 0 |
| Gleanlea Surgery | 4 | 4 | 0 |
| Haigh Hall medical centre | 1 | 1 | 0 |
| Healds road surgery | 3 | 2 | 1 |
| Mountain road surgery | 2 | 1 | 1 |
| New brewery lane surgery | 1 | 1 | 0 |
| Paddock surgery | 42 | 21 | 21 |
| Shipley medical practice | 4 | 3 | 1 |
| Sidings health centre | 2 | 1 | 1 |
| Thornton medical centre | 3 | 0 | 3 |
| West lodge Farsley | 8 | 3 | 5 |
| Willow medical practice | 7 | 4 | 3 |
| Windsor medical centre | 9 | 5 | 4 |

## Supplementary Table 2. Cardio-kidney-metabolic-pulmonary medications at baseline in the control and intervention group

|  | Intervention (n = 68) | Control (n = 70) |
| --- | --- | --- |
| Aspirin | 16 (23.5) | 15 (21.4) |
| ACE-i/ARB/ARNI | 36 (52.9) | 42 (60.0) |
| Beta blocker | 19 (27.9) | 17 (24.3) |
| Calcium channel blocker | 31 (45.6) | 28 (40.0) |
| Thiazide-like diuretic | 5 (7.4) | 10 (14.3) |
| Spironolactone | 3 (4.4) | 2 (2.9) |
| SGLT2 inhibitor | 9 (11.5) | 9 (12.9) |
| GLP-1RA +/- GIP agonist | 3 (4.4) | 3 (4.6) |
| Statins | 54 (79.4) | 52 (74.3) |
| Ezetimibe | 2 (2.9) | 6 (8.6) |
| PCSK9 inhibitors | 0 (0.0) | 0 (0.0) |
| Metformin | 7 (10.3) | 11 (15.7) |
| DPP4 inhibitors | 2 (2.9) | 1 (1.4) |
| Sulphonylurea | 1 (1.4) | 1 (1.4) |
| Insulin | 2 (2.9) | 2 (2.9) |
| Orlistat | 0 (0.0) | 0 (0.0) |
| Nicotine replacement therapy | 0 (0.0) | 0 (0.0) |
| Bupropion | 0 (0.0) | 0 (0.0) |
| Inhaler therapy | 7 (10.3) | 11 (15.7) |

Data are numbers (%) unless otherwise stated.

ACE-I, angiotensin converting enzyme inhibitor; ARB, angiotensin 2 receptor blocker; ARNI, angiotensin receptor-neprilysin inhibitor; DPP4, dipeptidyl peptidase 4; GLP-1 RA, glucagon-like peptide-1 receptor agonist; GIP, gastric inhibitory peptide; PCSK9, proprotein convertase subtilisin/kexin type 9; SGLT2, sodium glucose co-transporter 2;

## Supplementary Table 3. CKMP medication interventions by six months

| **CKMP therapy category** | **Therapy Initiation** | | **Therapy Increase** | |
| --- | --- | --- | --- | --- |
|  | Control arm | Intervention arm | Control arm | Intervention arm |
| Antihypertensive therapy | 6 | 9 | 5 | 5 |
| Glycaemic agent with CVD benefit | 2 | 6 | 0 | 1 |
| Glycaemic agent without CVD benefit | 1 | 2 | 0 | 0 |
| Lipid modification therapy | 7 | 19 | 1 | 10 |
| Obesity-specific treatment | 0 | 1 | 0 | 0 |
| Renin-angiotensin system inhibitor | 1 | 3 | 0 | 2 |
| Smoking cessation treatment | 0 | 0 | 0 | 0 |
| Respiratory inhaler therapy | 0 | 2 | 0 | 0 |

CKMP – cardio-kidney-metabolic-pulmonary; CVD, cardiovascular disease

## Supplementary Table 4. Reasons for non-adherence to CKMP medication recommendations

| **CKMP therapy category** | **Reason for non-adherence** | **Number (%)** |
| --- | --- | --- |
| Antihypertensive therapy | Intolerance/side effects | 1 (100) |
| Glycaemic agent with CVD benefit | Patient refusal  Alternative treatment  No longer indicated | 1 (25)  1 (25)  2 (50) |
| Glycaemic agent without CVD benefit | Alternative treatment  No details recorded | 2 (50)  2 (50) |
| Lipid modification therapy | Intolerance/side effects  Alternative treatment  Patient refusal  No details recorded | 3 (23.1)  2 (15.4)  3 (23.1)  5 (38.5) |
| Obesity-specific treatment | Did not qualify as per local guidelines  No longer indicated  No details recorded | 9 (81.8)  1 (9.1)  1 (9.1) |
| Renin-angiotensin system inhibitor | - | - |
| Smoking cessation treatment | No details recorded | 1 (100) |

Data are numbers (%) unless otherwise stated.

CKMP – cardio-kidney-metabolic-pulmonary; CVD, cardiovascular disease

## Supplementary Figure 1. Heat map of cardio-kidney-metabolic-pulmonary pharmacological interventions

## Supplementary Figure 2. Forest plot of new cardio-kidney-metabolic-pulmonary risk factor diagnoses

CKD, chronic kidney disease; COPD, chronic obstructive pulmonary disease; CKMP, cardio-kidney-metabolic-pulmonary.

# References

1.	Wu J, Nadarajah R, Nakao YM, Nakao K, Hogg D, Raveendra K, Arbel R, Haim M, Zahger D, Cowan C, Gale CP. Incident cardiovascular, renal, metabolic diseases and death in individuals identified for risk-guided atrial fibrillation screening: a nationwide cohort study. *Open Heart*. 2023;10. doi: 10.1136/openhrt-2023-002357
